# Comparison of the Physico-Mechanical and Weathering Properties of Wood–Plastic Composites Made of Wood Fibers from Discarded Parts of Pomelo Trees and Polypropylene

**DOI:** 10.3390/polym13162681

**Published:** 2021-08-11

**Authors:** Ke-Chang Hung, Wen-Chao Chang, Jin-Wei Xu, Tung-Lin Wu, Jyh-Horng Wu

**Affiliations:** 1 Department of Forestry, National Chung Hsing University, Taichung 402, Taiwan; d9833004@mail.nchu.edu.tw (K.-C.H.); ecsgunro@gmail.com (J.-W.X.); 2 Tainan District Agricultural Research and Extension Station, Council of Agriculture, Tainan 712, Taiwan; wcchang@mail.tndais.gov.tw; 3 College of Technology and Master of Science in Computer Science, University of North America, Fairfax, VA 22033, USA; tonywuwu22@gmail.com; 4 Department of Wood Science and Design, National Pingtung University of Science and Technology, Pingtung 912, Taiwan

**Keywords:** physico-mechanical property, polypropylene, pomelo tree, xenon arc accelerated weathering, wood–plastic composite (WPC)

## Abstract

The purpose of this study is to compare the characteristics of wood–plastic composites (WPCs) made of polypropylene (PP) and wood fibers (WFs) from discarded stems, branches, and roots of pomelo trees. The results show that the WPCs made of 30–60 mesh WFs from stems have better physical, flexural, and tensile properties than other WPCs. However, the flexural strengths of all WPCs are not only comparable to those of commercial wood–PP composites but also meet the strength requirements of the Chinese National Standard for exterior WPCs. In addition, the color change of WPCs that contained branch WFs was lower than that of WPCs that contained stem or root WFs during the initial stage of the accelerated weathering test, but the surface color parameters of all WPCs were very similar after 500 h of xenon arc accelerated weathering. Scanning electron microscope (SEM) micrographs showed many cracks on the surfaces of WPCs after accelerated weathering for 500 h, but their flexural modulus of rupture (MOR) and modulus of elasticity (MOE) values did not differ significantly during weathering. Thus, all the discarded parts of pomelo trees can be used to manufacture WPCs, and there were no significant differences in their weathering properties during 500 h of xenon arc accelerated weathering.

## 1. Introduction

As one of the most important families of fruits in the world, the large citrus family includes sweet oranges (*Citrus sinensis*), mandarins or tangerine oranges (*C. reticulata*), sour/bitter oranges (*C. aurantium*), lemons (*C. limon*), limes (*C. aurantifolia*), and grapefruit (*C. paradisi*), with global production numbering over 120 million tons per year [1,2]. Among the citrus species, the pomelo (*C. grandis* or *C. maxima*) is the largest citrus fruit of the Rutaceae family and is widely consumed in Taiwan [3]. In the Yunlin, Chiayi, and Tainan areas of Taiwan, pomelo cultivation covered a total area of approximately 1500 ha in 2015. In order to produce high-quality fruit, the pomelo tree must be pruned regularly [4]. However, pruning produces many useless twigs, branches, and stems that become agricultural waste, approximately 2250–13,500 tons per year. If this woody waste can be recycled and reused, it will increase the commercial value of local crops. The manufacture of value-added panels from wood–plastic composites (WPCs) could be one of the solutions to this problem of pomelo waste.

WPCs have been considered as alternatives to metals, plastics, and solid wood in automotive applications, aviation equipment components, sporting goods, and the construction industry (decking, fences, exterior wall panels, window frames, and roofing materials) [5,6,7,8,9,10]. In general, WPCs are manufactured by mixing wood particles or fibers as a reinforcement with a thermoplastic matrix under high temperature and pressure. Therefore, compared with inorganic fillers (e.g., mineral fillers and glass fibers) used in reinforced composites, WPCs show many advantages such as reducing the proportion and cost of the plastic matrix, increasing the stiffness of the plastic matrix, improving the physico-mechanical properties and processability of wood materials, renewability, low maintenance requirements, and environmentally friendliness [11,12,13,14,15,16,17,18,19]. In addition to wood particles or fibers, various agricultural wastes, such as bagasse [20,21], bamboo [16,18], cotton [11], coconut [22], hemp fiber [12], kenaf fiber [23], pineapple leaf [11], palm [9,24], rice husk [25], red pepper fruit stem [26], and straw [27], were also used as reinforcements or fillers for the thermoplastic composites. However, little information is available regarding the recycling and reuse of the pomelo waste. Furthermore, petrochemical-based thermoplastics including polyethylene (PE), polypropylene (PP), polyvinylchloride (PVC), and polystyrene (PS) are the most commonly used plastics for WPCs [28,29,30]. In the 21st century, biocomposites consisting of natural fiber and biodegradable polymer were an innovative idea, but petrochemical-based polymers are still the best engineering plastics [11]. Therefore, many studies are still focused on natural fiber reinforced petrochemical-based plastic composites. Among them, PP-based WPCs showed the strongest stiffness and bending strength [28,30].

Accordingly, the objective of the present study was to compare the physico-mechanical characteristics of WPCs made of PP and various sizes of wood fibers (WFs) from different discarded parts of pomelo trees. In addition, accelerated weathering is a powerful test for quality control and material certification. Therefore, in this study, the surface and flexural properties of WPCs were also evaluated after xenon arc accelerated weathering tests. To the best of our knowledge, this is the first comparative study concerning the weathering characteristics of WPCs containing WFs from different discarded parts of fruit trees.

## 2. Experimental

### 2.1. Materials

The discarded stems, branches, and roots of pomelo trees (*Citrus grandis* Osbeck cv. Matou Wentan) were kindly provided by the Tainan District Agricultural Research and Extension Station, Tainan County, Taiwan. WFs from the pomelo trees were prepared by hammer milling and sieving in order to obtain fibers within four size ranges: 16–20 mesh (1000–830 μm), 20–30 mesh (830–550 μm), 30–60 mesh (550–250 μm), and <60 mesh (<250 μm). Polypropylene pellets (PP, Globalene 7633), purchased from LCY Chemical Co., (Taipei, Taiwan), had a density of 896 kg/m^3^, a melt flow index (MFI) of 2 g/10 min, and a melting point of 170 °C. Commercially available malleated PP (MAPP, maleic anhydride: 8–10 wt%; density: 934 kg/m^3^; melting point: 156 °C; MFI: 115 g/10 min) used as a coupling agent was purchased from Sigma-Aldrich Chemical Co., (St. Louis, MO, USA).

### 2.2. Preparation of the Composite Panels

As presented in Table 1, six kinds of WFs from different discarded parts of pomelo trees with various sizes were used to manufacture WPCs. The sample codes of various WPCs were described as WPC_XYY_, where X represents the WFs from the discarded parts of pomelo trees (B: branch; R: root; S: stem), and YY is the maximum size (mesh) of WFs used in the given composite. The weight ratio of oven-dried WFs (moisture content < 3%), PP and MAPP was 50/47/3 (wt%) for WPCs. WPCs were compounded at 200 °C for 10 min by a YKI-3 Banbury mixer (Goldspring Enterprise Inc., Taichung, Taiwan) at a rotor speed of 50 rpm. After compounding, the mixtures were extruded and pellets. The expected density of the WPCs was 1000 kg/m^3^. The pellets were used to form WPC mats with dimensions of 300 mm × 200 mm. Then, 3 mm thick plate samples were compression molded in a flat-platen process according to our previous reports [19,29,31,32,33,34,35]: (1) hot-pressing at 200 °C and 2.5 MPa for 5 min; (2) finishing on cold pressing until the temperature of the WPCs dropped to 40 °C (approximately 5 min).

### 2.3. Xenon Arc Accelerated Weathering Test

Accelerated weathering tests were carried out in a Q-SUN Xe-3 xenon arc chamber (Q-Lab Co., Westlake, OH, USA) according to the cycle 1 exposure condition of ASTM G 155-13 standard [36]. The exposure cycle consisted of 102 min of irradiation (with an average irradiance of 0.35 W/m^2^ at 340 nm) at a black panel temperature of 63 °C, followed by 18 min of light and water spray (air temperature not controlled). The total exposure time was 500 h, and the flexural properties and surface characteristics of the samples were regularly measured during the exposure period.

### 2.4. Characterizations of Composite Properties

The density and moisture content of the WPCs were determined according to ASTM D2395-07a [37] and ASTM D4442-07 [38]. Flexural tests were carried out according to ASTM D790-09 [39]. Specimens with dimensions of 60 mm × 13 mm × 3 mm were used to evaluate the modulus of rupture (MOR) and modulus of elasticity (MOE) by a three-point static bending test at a loading speed of 1.28 mm/min and span of 48 mm. In addition, tensile properties were measured for ASTM D638-08 type I specimens (dog-bone specimens) at a tensile speed of 5 mm/min [40]. All samples were conditioned at 20 °C and 65% relative humidity for two weeks prior to testing, and at least five replicate specimens were tested for each formulation.

### 2.5. ATR-FTIR Spectral Measurement

Attenuated total reflectance Fourier transform infrared (ATR-FTIR) spectra of the WPCs were recorded on a Spectrum 100 FTIR spectrometer (Perkin–Elmer, Buckinghamshire, UK) equipped with a deuterated triglycine sulfate (DTGS) detector and a MIRacle ATR accessory (Pike Technologies, Madison, WI, USA). The spectra were collected by co-adding 32 scans at a resolution of 4 cm^−1^ in the range from 650 to 4000 cm^−1^. Five spectra were acquired at room temperature for each composite. The carbonyl index (CI) was calculated by using the equation CI = *I*_1712 cm^−1^_/*I*_2918 cm^−1^_, where *I* represents the peak intensity. The peak intensity was normalized by using the peak at 2918 cm^−1^, which corresponds to asymmetric C–H stretching vibrations of methylene groups (–CH_2_–) in PP [28,29,41]. Additionally, the change ratio of CI (CROCI) during weathering was subsequently calculated as follows: CROCI (%) = (CI_w_/CI_0_) × 100, where CI_0_ and CI_w_ are the CI values of WPCs before and after xenon arc accelerated weathering, respectively.

### 2.6. Measurement of Surface Color

The color parameters of the composite surface were measured by a UV-Vis spectrophotometer (Lambda 850+, Perkin–Elmer, Waltham, MA, USA) equipped with a 60 mm diameter PbS integrating sphere (Perkin–Elmer, Waltham, MA, USA) and a 20 mm diameter test window. The color parameters L*, a*, and b* of all specimens were obtained directly from the colorimeter. Based on the CIE L*a*b* color system, L* is the value on the white/black axis, a* is the value on the red/green axis, b* is the value on the yellow/blue axis, and the ΔE* value is the color difference (ΔE* = [(ΔL*)^2^ + (Δa*)^2^ + (Δb*)^2^]^1/2^).

### 2.7. Scanning Electron Microscopy

Scanning electron microscopy (SEM) was used to examine the surface characteristics of WPCs after the xenon arc accelerated weathering. The specimens were dried and then sputtered with gold before SEM analysis. A JEOL JSM-6330F scanning electron microscope (Tokyo, Japan) with a field emission gun and the accelerating voltage of 2.8 kV was used to collect SEM images of the composite specimen.

### 2.8. Analysis of Variance

All the results were expressed as the mean ± standard deviation (SD). The significance of differences was calculated by Scheffe’s test or Student’s *t*-test, and *p* values < 0.05 were considered to be significant.

## 3. Results and Discussion

### 3.1. The Physical and Flexural Properties of the WPCs

**Figure 1:**
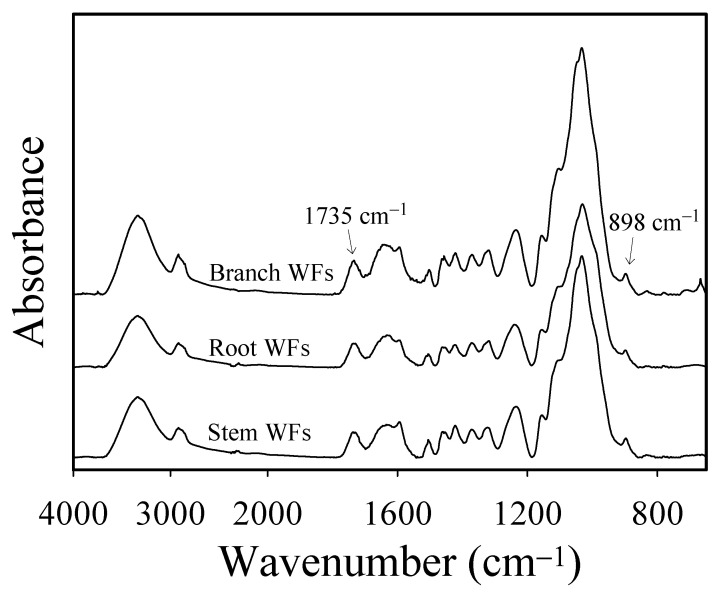
ATR-FTIR spectra of WFs from various pomelo tree parts.

The physical and flexural properties of the WPCs that contained WFs with different sizes from various parts of pomelo trees are listed in Table 1. For a certain WF size of 20–30 mesh, the densities of WPCs with branch WFs (WPC_B20_), root WFs (WPC_R20_), and stem WFs (WPC_S20_) were approximately 1067–1076 kg/m^3^, and there were no significant differences among them. However, the moisture content of WPC_B20_ (2.77%) was higher than that of WPC_R20_ (1.00%) and WPC_S20_ (1.01%), but this value was similar to that moisture content (2.4%) reported by Yang et al. [17] for wood/recycled-HDPE composites (50/50 wt%). A possible explanation for this observation is that the juvenile wood content of young branches was higher than that of roots and stems. Juvenile wood has a lower density and higher hemicellulose content compared to mature wood [42], which results in greater hygroscopicity for the WPCs made of branch WFs than the WPCs made of WFs from roots and stems. Figure 1 shows the ATR-FTIR spectra of pomelo branch, root, and stem WFs. The spectra clearly confirmed that the intensity of the C=O stretching band (i.e., acetyl groups in hemicellulose) of branch WFs at 1735 cm^−1^ was obviously higher than that of root and stem WFs. The intensity ratios of the peaks at 1735 cm^−1^ and 898 cm^−1^ for branch, root, and stem WFs were 1.36, 1.24, and 1.25, respectively. In addition, the WPCs with branch WFs also exhibited the worst flexural properties among all WPCs. The MOR and MOE of WPC_B20_ were 39 MPa and 2.2 GPa, respectively. Nevertheless, the strength of WPC_B20_ was comparable to that of commercial wood–PP composites (36.5–42.7 MPa flexural strength) reported by Klyosov [43], and it also met the strength requirement (exceeding 20 MPa) of exterior WPCs (types EX I and II) in accordance with the Chinese National Standard CNS 15,730 [44]. Meanwhile, the flexural properties of WPC_B20_ were higher than that reported by Lazarini and Marconcini [21] for PP-based composites containing 50 wt% bagasse fibers (25 MPa of MOR and 2.0 GPa of MOE). Generally, the strength was lower for juvenile wood, and the fibers were shorter for juvenile wood than mature wood [42], which might cause the flexural strength of WPC_B20_ to be less than that of WPC_R20_ and WPC_S20_. However, the tensile properties of WPC_S20_ and WPC_B20_ were better than those of WPC_R20_. This result implied that not only the characteristics of the WFs but also the interfacial adhesion between the WFs and polymeric matrix affected the mechanical properties of the WPCs. According to the above results, WPC_S20_ showed the best flexural and tensile strength among the WPCs that contained branch, root, and stem WFs. Therefore, the stem WFs were subsequently used as natural fillers to investigate the influence of WF size on the physical, flexural, and tensile properties of WPCs.

Table 1 shows that there were no significant differences in the density among all WPCs with different WF sizes, and the density values were in the range from 1073 to 1082 kg/m^3^. In addition, the moisture content of the WPCs decreased with decreasing WF size, and the WPCs with less than 60 mesh WFs (WPC_S60_) exhibited the lowest moisture content (0.90%). This trend is similar to that reported by Rahman et al. [45] for composites made of recycled polyethylene terephthalate (PET) and different sizes of WFs. Rahman et al. [45] found that there were more voids in the WPCs that contained larger WFs, which made it easier for moisture to penetrate through the openings of the composites. Moreover, the WPCs with the smallest WFs (WPC_S60_) showed the lowest MOR (39 MPa) and MOE (2.3 GPa) values among WPCs with different sizes of WFs. A similar result was also observed in studies by Rahman et al. [45] and Chen et al. [46]. However, the tensile properties of WPC_S16_ were lower than those of other WPCs. The tensile strength, tensile modulus, and elongation at break of WPC_S16_ were 21.9 MPa, 2.27 GPa, and 1.6%, respectively. These results are consistent with the results reported by Ashori and Nourbakhsh [47], Feng et al. [48], and Onuoha [49] for the changes in tensile properties with WF sizes. A possible reason is that the smaller WFs were dispersed more thoroughly and provided a larger contact area with the PP matrix, thereby promoting better interfacial adhesion and lower stress concentrations [45,48,49]. On the other hand, large WFs had rougher surfaces and looser structures, which resulted in poorer compatibility between the PP matrix and WFs. Therefore, it was easy to form the abovementioned void defects, resulting in stress concentrations and relatively weak tensile properties during tensile tests. Accordingly, WPC_S30_ possesses better physical, flexural, and tensile properties than the other WPCs. Thus, the optimal size of pomelo WFs for manufacturing WPCs is 30–60 mesh, according to this study.

### 3.2. Characteristics of the WPCs during Xenon Arc Accelerated Weathering

#### 3.2.1. Color Changes of the WPCs during Accelerated Weathering

**Figure 2:**
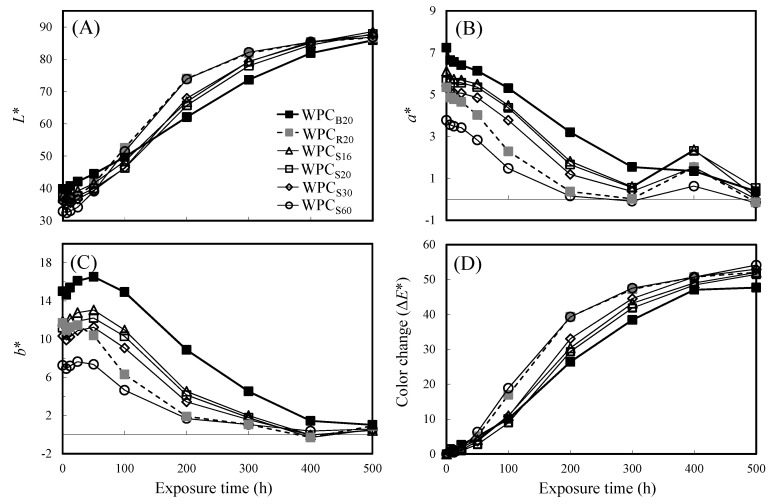
The color change of various WPCs during xenon arc accelerated weathering. (**A**) L*, (**B**) a*, (**C**) b*, and (**D**) ΔE* values. Each reported value is the average of five replicate specimens for each formulation.

The color variations of the WPCs that contain WFs with different sizes and sources (parts of pomelo trees) during 500 h of xenon arc accelerated weathering were evaluated by the CIE L*a*b* color system. As shown in Figure 2A, the L* values of all WPCs were in the range from 32.9 to 39.9 before accelerated weathering, and the WPC_B20_ and WPC_S60_ exhibited the highest and lowest L* values, respectively. The L* values were lower than the results of Hung et al. [32] and Stark [50], who reported that the L* values were 64.3 and 50–70, respectively. However, the L* values were similar to the results (approximately 40) reported by Stark and Matuana [51]. In addition, the L* value of all WPCs increased with increasing exposure time during 400 h of accelerated weathering and then leveled off. This result is similar to that of Stark [50] and Stark and Matuana [51], who reported that the colors of wood flour‒plastic composites became lighter during accelerated weathering, which was mainly due to bleaching of the wood component. Among these WPCs, the changes in the L* values of WPC_R20_ and WPC_S60_ were similarly higher compared to those of the other WPCs during 100–400 h of weathering, and the L* changes were smallest for WPC_B20_. However, after 500 h of accelerated weathering, the L* values of all WPCs were approximately 85.9–88.6, and there were no significant differences among them. These values were very similar to those reported by Stark [50] and Stark and Matuana [51]. In addition, the changes in the a* and b* values of all WPCs followed similar trends (Figure 2B,C), but WPC_B20_ and WPC_S60_ had the highest and the lowest values, respectively. After 500 h of accelerated weathering, the a* and b* values of all WPCs dropped from 3.8–7.2 and 7.2–15.0 to −0.2–0.5 and 0.4–1.0, respectively. These results indicate that the surface colors of all WPCs faded and became gray-white. According to a report by Kanbayashi et al. [52], most of the lignin bands disappeared from the Raman spectrum for Japanese beech (*Fagus crenata* Blume) after 500 h of accelerated weathering, indicating that the lignin photodegraded and leached out of the wood. At the same time, the transparency of the plastic on the surfaces of WPCs declined after weathering, which may be associated with secondary crystallization processes induced by short macromolecular chains resulting from amorphous polymer chain cleavage during UV weathering [53]. Therefore, the WPCs lost their woody brown color and became gray-white during weathering. These results are consistent with other WPC weathering studies [29,54,55,56].

Meanwhile, Figure 2D shows that the color change of all WPCs increased with increasing exposure times until 400 h of accelerated weathering and then leveled off. Of these WPCs, WPC_R20_ and WPC_S60_ exhibited greater color changes during the initial stage of accelerated weathering. The ΔE* values of WPC_R20_ (39.4) and WPC_S60_ (39.4) were higher than those of other WPCs after weathering for 200 h. However, the ΔE* values of all WPCs were very close after 500 h of accelerated weathering. For WPC_R20_, WPC_S16_, WPC_S20_, WPC_S30_, and WPC_S60_, the ΔE* values were 52.0, 52.0, 51.5, 53.0, and 54.1, respectively. WPC_B20_ showed the smallest color change (ΔE* = 47.7) during 500 h of accelerated weathering. Accordingly, the color change rate of WPCs with branch WFs was lower than that of other WPCs, although the surface color parameters were similar after accelerated weathering for 500 h.

#### 3.2.2. Surface and Flexural Properties of the WPCs during Accelerated Weathering

**Figure 3:**
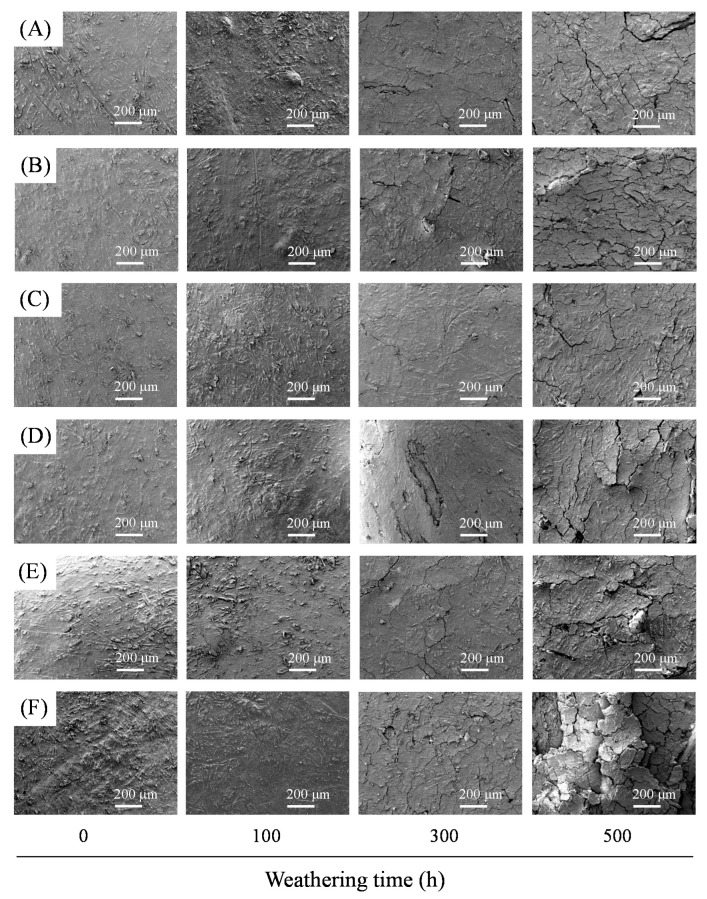
SEM micrographs of various WPCs before and after xenon arc accelerated weathering for 100, 300, and 500 h. (**A**) WPC_B20_, (**B**) WPC_R20_, (**C**) WPC_S16_, (**D**) WPC_S20_, (**E**) WPC_S30_, and (**F**) WPC_S60_.

Figure 3 shows SEM micrographs of WPCs after xenon arc accelerated weathering for 0, 100, 300, and 500 h. Before accelerated weathering, the surfaces of all WPCs were smooth, and the WFs were covered with the polymer. However, some random cracks were observed on the surfaces of all WPCs after accelerated weathering for 300 h, and the amount and size of the crazing increased as functions of weathering time. These findings are in agreement with reports by Fabiyi et al. [56] and Turku et al. [57]. They pointed out that photooxidation causes polymer chain scission, resulting in cracking of highly crystallized polymer zones and/or differential contraction between the surface and interior sections during accelerated weathering. Furthermore, WPCs with WFs from different pomelo tree parts showed the same surface characteristics, but the WPCs with finer WFs exhibited additional crack formation after 500 h of accelerated weathering compared to those with coarser WFs. A possible explanation for this result is that finer WFs have larger surface areas, resulting in a more significant differential contraction between the WFs and PP matrix than coarser WFs in other WPCs. 

Table 2 shows the changes in flexural properties of various WPCs during 500 h of xenon arc accelerated weathering. The results showed that there was no significant difference in the flexural properties among all weathered WPCs. The MOR and MOE values were in the ranges of 41–45 MPa and 2.4–2.8 GPa, respectively. This result is similar to that of Beg and Pickering [58], who reported that the mechanical properties of PP and its wood fibre composites did not notably change during accelerated weathering for 600 h. Accordingly, the flexural strengths of these weathered WPCs are still comparable to those of commercial wood–PP composites, and they meet the requirements of Chinese National Standard CNS 15,730 for exterior WPCs (types EX I and II).

#### 3.2.3. ATR-FTIR Analysis of the WPCs during Accelerated Weathering

**Figure 4:**
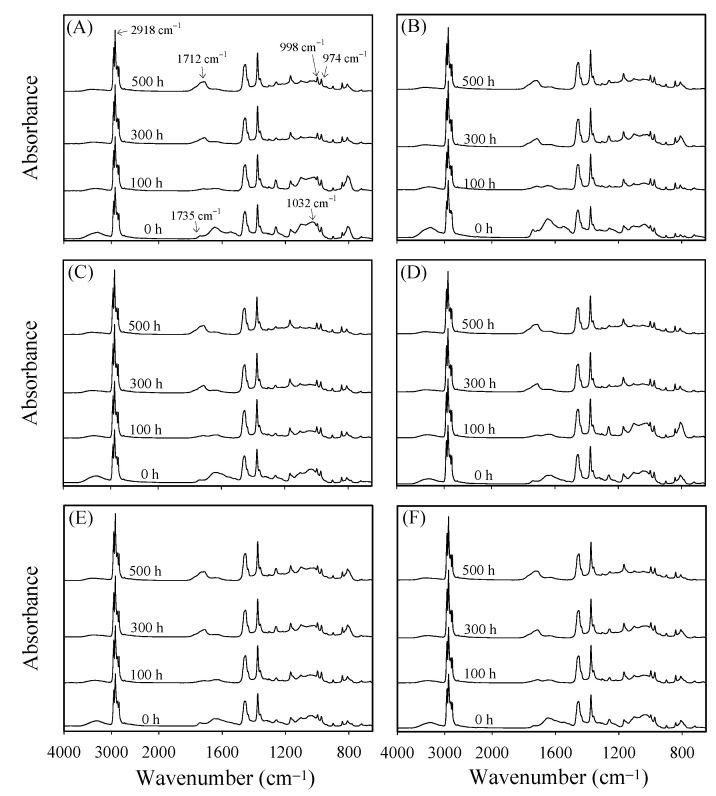
ATR-FTIR spectra of various WPCs before and after xenon arc accelerated weathering for 100, 300, and 500 h. (**A**) WPC_B20_, (**B**) WPC_R20_, (**C**) WPC_S16_, (**D**) WPC_S20_, (**E**) WPC_S30_, and (**F**) WPC_S60_.

In order to understand the chemical changes on the WPC surfaces during accelerated weathering, ATR-FTIR spectroscopy was used. Figure 4 shows that the bands at 1032, 1735, and 3050–3600 cm^−1^ were assigned to the C–O–C groups of cellulose and hemicellulose [55,56], C=O stretching in acetyl groups of hemicellulose [32,55], and hydroxyl groups of cellulose [55,56] before accelerated weathering, respectively. The intensities of these bands of all WPCs were lower after accelerated weathering. Fabiyi and McDonald [56] observed similar results, and they pointed out that wood fibers degraded and leached from the surface of WPC during weathering. On the other hand, the chain scission of PP can be caused by photodegradation via Norrish I and II reactions during weathering, as indicated by increases in the concentrations of carbonyl and vinyl groups [29,32]. 

**Figure 5:**
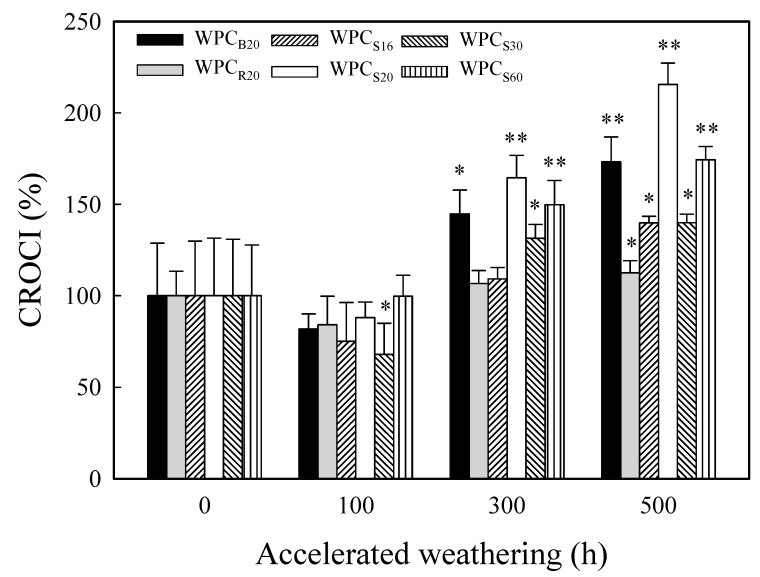
The CROCI values of various WPCs before and after xenon arc accelerated weathering for 100, 300, and 500 h. Values are mean ± SD (*n* = 5). *: *p* < 0.05; **: *p* < 0.01 (one-tailed test) compared to the unweathered WPC.

As shown in Figure 4, after accelerated weathering for more than 300 h, a new broad carbonyl band was present at 1680–1800 cm^−1^, but the band for the vinyl group (1640 cm^−1^) did not increase significantly. A similar result was also reported by Stark and Matuana [59]. This result indicated that the photodegradation of PP occurred mainly through Norrish I reactions. Ndiaye et al. [60] reported that three newly formed carbonyl groups were observed in weathered PP-based WPCs, including carboxylic acids (1712 cm^−1^), esters (1735 cm^−1^), and γ-lactone (1780 cm^−1^). Of these three types of carbonyl groups, carboxylic acids exhibited the strongest intensity. Therefore, in this study, the carbonyl index (CI) was calculated by the intensity ratio of absorption at 1712 cm^−1^ compared to that at 2918 cm^−1^ (asymmetric CH_2_ stretching vibration band of PP) in order to evaluate the degree of photooxidation of the PP matrix. The greater the CI value, the higher the degree of photooxidation. Figure 5 shows the variations in the change ratio of CI (CROCI) values for various WPCs during 500 h of accelerated weathering. The CROCI values were affected by the degradation of hemicellulose (with an absorption peak at approximately 1735 cm^−1^ that overlapped those of the carboxylic acids), and the CROCI value of all WPCs slightly decreased during the early stage of weathering (100 h). However, the CROCI values of all WPCs increased afterward with increasing accelerated weathering time due to the formation of photooxidative carboxylic acids. A similar increasing trend was also found in other studies [55,56,60]. The CROCI values increased in the order of WPC_S20_, WPC_S60_, WPC_B20_, WPC_S16_, WPC_S30_, and WPC_R20_ by 216 ± 12, 174 ± 7, 173 ± 14, 140 ± 4, 140 ± 5, and 113 ± 7%, respectively, after accelerated weathering for 500 h. Among them, WPC_R20_ seems to show the best photostability, and the reason for this stability needs to be further investigated in the future.

**Figure 6:**
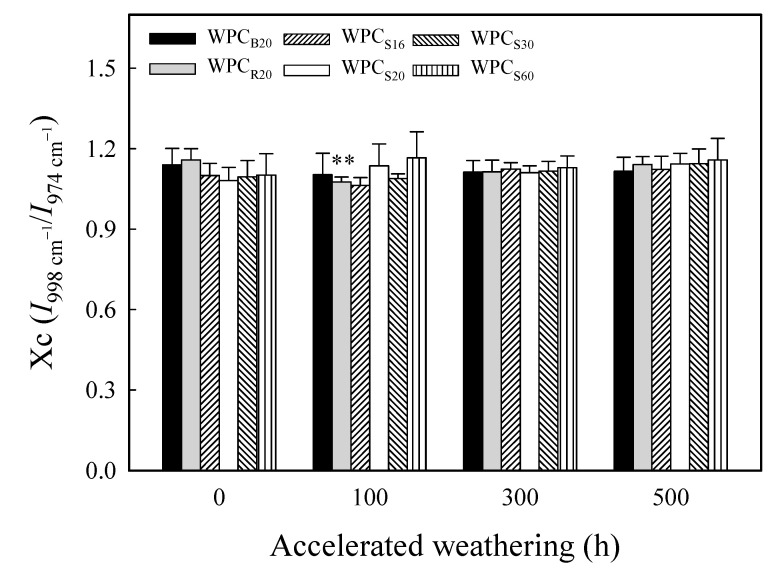
The Xc values of various WPCs before and after xenon arc accelerated weathering for 100, 300, and 500 h. Values are the mean ± SD (*n* = 5). **: *p* < 0.01 (one-tailed test) compared to the unweathered WPC.

On the other hand, Jabarin and Lofgren [61] and Zou et al. [62] reported that photooxidation causes chain scission in the amorphous phase of polyolefins during the initial weathering period, and the resulting shorter molecules are believed to possess sufficient chain mobility to cause secondary crystallization. Moreover, the depth of weathering degradation caused by UV irradiation of WPCs varied from 200 to 2540 μm [52]. In Figure 4, the band at 998 cm^−1^ was assigned to the C–H bending of crystalline phase PP, and the ratio of the intensities at 998 cm^−1^ and 974 cm^−1^ was considered a linearly proportional measure of the degree of crystallinity [63]. Therefore, the value of *I*_998 cm^−1^_/*I*_974 cm^−1^_ (Xc) was used as an index for investigating the change in crystallinity of the PP matrix during xenon arc accelerated weathering. Figure 6 shows that the Xc values of all WPCs were in the range from 1.06 to 1.17 during 500 h of accelerated weathering, and there was no significant difference among them. In other words, the matrix crystallinity of all WPCs did not change after accelerated weathering for 500 h. The possible reasons are that the accelerated weathering time was too short and the photooxidized fragments of the PP matrix leached from the composites.

## 4. Conclusions

Wood–plastic composites (WPCs) were successfully made of polypropylene and waste pomelo wood fibers (WFs). In this study, the WPCs with 30–60 mesh stem WFs (WPC_S30_) had a lower moisture content and the best flexural and tensile properties. The ATR-FTIR results showed that a new broad carbonyl band formed, but the absorption by the vinyl group did not increase significantly after xenon arc accelerated weathering for 500 h, indicating that PP underwent photodegradation mainly through Norrish I reactions. The surface of all weathered WPCs showed observable color changes and cracking, but the matrix crystallinity and flexural properties did not change notably. The flexural strength of all WPCs was comparable to those of commercial wood–PP composites. Accordingly, all the woody parts of discarded pomelo trees can be used as natural reinforcements for exterior thermoplastic composites. 

## Figures and Tables

**Table 1:** Effect of pomelo tree parts and WF sizes on the physical, flexural, and tensile properties of WPCs.

Code	Part	WF Size (mesh)	Density (kg/m^3^)	Moisture Content (%)	Flexural Properties		Tensile Properties		
					MOR (MPa)	MOE (GPa)	Tensile Strength (MPa)	Tensile Modulus (GPa)	Elongation at Break (%)
WPC_B20_	Branch	20–30	1067 ± 10 ^A^	2.77 ± 0.08 ^A^	39 ± 2 ^B^	2.2 ± 0.1 ^C^	23.0 ± 0.5 ^B^	2.38 ± 0.05 ^A^	1.9 ± 0.1 ^A^
WPC_R20_	Root	20–30	1070 ± 13 ^A^	1.00 ± 0.08 ^B^	43 ± 3 ^A^	2.7 ± 0.2 ^A^	20.1 ± 0.5 ^C^	2.22 ± 0.08 ^C^	1.3 ± 0.1 ^B^
WPC_S16_	Stem	16–20	1078 ± 17 ^a^	1.01 ± 0.05 ^a^	43 ± 3 ^a^	2.5 ± 0.2 ^ab^	21.9 ± 1.0 ^c^	2.27 ± 0.08 ^b^	1.6 ± 0.2 ^c^
WPC_S20_	Stem	20–30	1076 ± 14 ^aA^	1.01 ± 0.04 ^aB^	42 ± 3 ^aA^	2.4 ± 0.2 ^bB^	23.4 ± 0.4 ^bA^	2.30 ± 0.09 ^abB^	1.9 ± 0.1 ^bA^
WPC_S30_	Stem	30–60	1073 ± 16 ^a^	0.96 ± 0.04 ^ab^	44 ± 2 ^a^	2.6 ± 0.1 ^a^	24.1 ± 0.2 ^a^	2.35 ± 0.08 ^a^	2.1 ± 0.2 ^a^
WPC_S60_	Stem	<60	1082 ± 14 ^a^	0.90 ± 0.14 ^b^	39 ± 3 ^b^	2.3 ± 0.1 ^b^	23.3 ± 0.4 ^b^	2.28 ± 0.07 ^ab^	2.0 ± 0.2 ^ab^

Values are the mean ± SD (*n* = 15). Different capital and lowercase letters within a column indicate significant differences among WPCs with various tree parts and sizes of wood fibers (*p* < 0.05), respectively.

**Table 2:** The flexural properties of various WPCs after xenon arc accelerated weathering for 100, 300, and 500 h.

Code	Part	WF Size (mesh)	MOR (MPa)			MOE (GPa)		
			100 h	300 h	500 h	100 h	300 h	500 h
WPC_B20_	Branch	20–30	42 ± 1 ^B^	42 ± 3 ^A^	43 ± 3 ^A^	2.6 ± 0.2 ^A^	2.6 ± 0.2 ^A^	2.5 ± 0.2 ^AB^
WPC_R20_	Root	20–30	47 ± 1 ^A^	45 ± 2 ^A^	45 ± 3 ^A^	2.9 ± 0.2 ^A^	2.8 ± 0.2 ^A^	2.8 ± 0.3 ^A^
WPC_S16_	Stem	16–20	47 ± 2 ^a^	45 ± 2 ^a^	42 ± 4 ^a^	2.8 ± 0.1 ^a^	2.6 ± 0.2 ^a^	2.4 ± 0.2 ^a^
WPC_S20_	Stem	20–30	48 ± 1 ^aA^	44 ± 1 ^aA^	41 ± 2 ^aA^	2.8 ± 0.1 ^aA^	2.6 ± 0.1 ^aA^	2.4 ± 0.2 ^aB^
WPC_S30_	Stem	30–60	44 ± 3 ^a^	42 ± 2 ^a^	44 ± 2 ^a^	2.8 ± 0.1 ^a^	2.6 ± 0.1 ^a^	2.5 ± 0.2 ^a^
WPC_S60_	Stem	<60	45 ± 4 ^a^	44 ± 2 ^a^	45 ± 1 ^a^	2.8 ± 0.3 ^a^	2.7 ± 0.1 ^a^	2.7 ± 0.1 ^a^

Values are the mean ± SD (*n* = 5). Different capital and lowercase letters within a column indicate significant differences among WPCs with various tree parts and sizes of wood fibers (*p* < 0.05), respectively.

## Data Availability

The data presented in this study are available on request from the corresponding author.
